# Relative validity of a mobile AI-technology–assisted dietary assessment in adolescent females in Vietnam

**DOI:** 10.1093/ajcn/nqac216

**Published:** 2022-08-09

**Authors:** Phuong Hong Nguyen, Lan Mai Tran, Nga Thu Hoang, Duong Thuy Thi Trương, Trang Huyen Thi Tran, Phuong Nam Huynh, Bastien Koch, Peter McCloskey, Rohit Gangupantulu, Gloria Folson, Boateng Bannerman, Alejandra Arrieta, Bianca C Braga, Joanne Arsenault, Annalyse Kehs, Frank Doyle, David Hughes, Aulo Gelli

**Affiliations:** International Food Policy Research Institute, Washington, DC, USA; Thai Nguyen University of Pharmacy and Medicine, Thai Nguyen, Vietnam; Emory University, Atlanta, GA, USA; National Institute of Nutrition, Hanoi, Vietnam; Thai Nguyen University of Pharmacy and Medicine, Thai Nguyen, Vietnam; Thai Nguyen University of Pharmacy and Medicine, Thai Nguyen, Vietnam; National Institute of Nutrition, Hanoi, Vietnam; International Food Policy Research Institute, Washington, DC, USA; Penn State University, State College, PA, USA; Penn State University, State College, PA, USA; University of Ghana, Accra, Ghana; University of Ghana, Accra, Ghana; International Food Policy Research Institute, Washington, DC, USA; Friedman School of Nutrition Policy and Science, Tufts University, Boston, MA, USA; Intake–Center for Dietary Assessment, FHI Solutions, Washington, DC, USA; Penn State University, State College, PA, USA; Penn State University, State College, PA, USA; Penn State University, State College, PA, USA; International Food Policy Research Institute, Washington, DC, USA

**Keywords:** adolescent, AI-assisted dietary assessment, app, 24-hour recall, food weight record, smartphone, relative validity, Vietnam

## Abstract

**Background:**

There is a gap in data on dietary intake of adolescents in low- and middle-income countries (LMICs). Traditional methods for dietary assessment are resource intensive and lack accuracy with regard to portion-size estimation. Technology-assisted dietary assessment tools have been proposed but few have been validated for feasibility of use in LMICs.

**Objectives:**

We assessed the relative validity of FRANI (Food Recognition Assistance and Nudging Insights), a mobile artificial intelligence (AI) application for dietary assessment in adolescent females (*n* = 36) aged 12–18 y in Vietnam, against a weighed records (WR) standard and compared FRANI performance with a multi-pass 24-h recall (24HR).

**Methods:**

Dietary intake was assessed using 3 methods: FRANI, WR, and 24HRs undertaken on 3 nonconsecutive days. Equivalence of nutrient intakes was tested using mixed-effects models adjusting for repeated measures, using 10%, 15%, and 20% bounds. The concordance correlation coefficient (CCC) was used to assess the agreement between methods. Sources of errors were identified for memory and portion-size estimation bias.

**Results:**

Equivalence between the FRANI app and WR was determined at the 10% bound for energy, protein, and fat and 4 nutrients (iron, riboflavin, vitamin B-6, and zinc), and at 15% and 20% bounds for carbohydrate, calcium, vitamin C, thiamin, niacin, and folate. Similar results were observed for differences between 24HRs and WR with a 20% equivalent bound for all nutrients except for vitamin A. The CCCs between FRANI and WR (0.60, 0.81) were slightly lower between 24HRs and WR (0.70, 0.89) for energy and most nutrients. Memory error (food omissions or intrusions) was ∼21%, with no clear pattern apparent on portion-size estimation bias for foods.

**Conclusions:**

AI-assisted dietary assessment and 24HRs accurately estimate nutrient intake in adolescent females when compared with WR. Errors could be reduced with further improvements in AI-assisted food recognition and portion estimation.

## Introduction

Unhealthy diets are estimated to cause 20% of global mortality (∼11 million) (1). Recent trends involving increased consumption of unhealthy foods and reductions in physical activity have contributed to increases in rates of overweight and obesity (2). Data on food and nutrient consumption are essential to inform nutrition policies and programs. However, there are important gaps in the data on diets in low- and middle-income countries (LMICs), particularly for school-age children and adolescents (3). Adolescence is a sensitive time to form habits and shape decisions on food choice, which can influence the rapid physical and psychosocial growth and development (4 , 5); thus, dietary data from adolescents are particularly important.

Collection and use of individual-level, quantitative dietary intake data have long been hindered due to bottlenecks related to high cost, time burden, complexity, and limited technical capacity (6). Among several methods to collect individual-level dietary data, dietary surveys commonly use the multi-pass 24-h recall (24HR) method that has been validated for use in LMICs in adults self-reporting their intake or that of their young children (7), and to some degree in adolescents (8). However, the age at which children and adolescents can accurately self-report food intake without caregiver assistance is not clear and a range of respondent- and observer-related issues are known to vary with age, including the ability and willingness to self-report intake and the variability in daily nutrient intakes (9). Tailoring the dietary assessment method with respondent characteristics is paramount. Technology-assisted dietary assessment tools including remote food photography methods (10) have been proposed in some studies, but existing tools are constrained by lack of assessments of validity and feasibility of use in LMICs, including in adolescents (6).

The Nudging for Good project is aimed at developing and examining the feasibility of using innovative artificial intelligence (AI) mobile technology to provide real-time diagnostics and tailored “nudging” on dietary intake as a strategy to improve diets and nutrition of adolescent females living in urban settings in Ghana and Vietnam (11). This project involves an interdisciplinary collaboration between the International Food Policy Research Institute, Penn State/FAO, the University of Ghana, the National Institute of Nutrition, and the Thai Nguyen National Hospital in Vietnam. The intervention design has 3 main stages. Briefly, the first stage focused on preparing a food database and image library including the following: *1*) developing a food inventory with priority foods; *2*) preparing, cooking, and taking graduated pictures of foods; and *3*) annotating the foods in the pictures and linking to the food database. In the second stage, the annotated pictures were used to train a semantic segmentation AI model for recognizing food and estimating portion sizes. In the third stage, the Food Recognition Assistance and Nudging Insights (FRANI) mobile app was developed including *1*) conducting formative research (2 rounds of focus group discussions) with users to develop user interface and *2*) developing an Android-based mobile phone application integrating AI-model and user interaction (12).

This study is aimed at evaluating the relative validity of FRANI, the new mobile AI application for dietary assessment in adolescent females aged 12–18 y in Vietnam against the gold standard of weighed records (WR) and comparing the performance of FRANI with a standard 24HR method. Specifically, the study objectives included the following: *1*) estimating nutrient and the adequacy of micronutrient intake using the 3 methods, *2*) assessing the equivalence bounds and extent of agreement with WR for FRANI and 24HR methods, and *3*) examining sources of error for FRANI and 24HR methods.

## Methods

### Study design, participants, and setting

The study was conducted in Vietnam, a Southeast Asian lower-middle-income country that has undergone a nutrition transition in food supply, food prices, household food expenditures, diets, and nutrition outcomes in the last few decades (13). Participants were recruited from urban communities in Thai Nguyen, a city in the northern province of Vietnam. Adolescents were eligible for inclusion if they met the following criteria: aged 12–18 y, capable of using smartphones with the FRANI app (provided by the project), and willingness to use it for 1 wk, allowing enumerators to shadow them for 3 d to conduct WR, and willingness to participate in three 24HR sessions. A total of 36 adolescent females aged 12–18 y were recruited in 5 different areas of the city on a voluntary basis. The intended sample size was based on the ability to detect a 10% difference in energy intake in the different dietary assessment methods and detecting equivalence within 10% bounds (ɑ = 0.05, B = 20%), as shown in a validation study in a similar study population (8). Recruitment was conducted by visiting identified adolescents at their homes 1 wk before the intended research day. Field enumerators met with adolescents and caregivers, presented the study's purpose and procedures, sought informed consent by parents and assent by children, and made appointments for the data collection week.

### Dietary assessment

**FIGURE 1:**
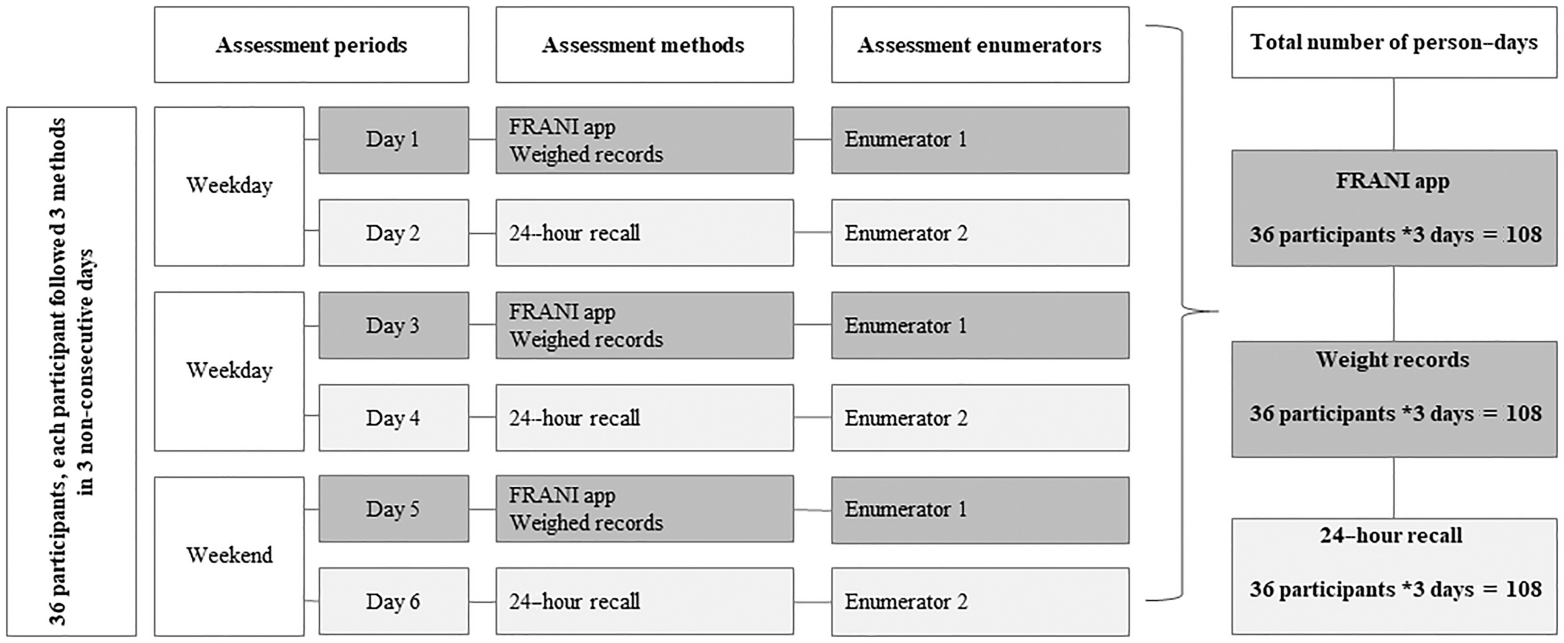
Participant flow chart. FRANI, Food Recognition Assistance and Nudging Insights.

Dietary intake was assessed on 3 nonconsecutive days, including 2 weekdays and a day on a weekend using 3 methods: mobile FRANI app, WR, and 24HR (**Figure 1**). The reference days for the 3 methods were the same and therefore directly comparable. Data collection for the WR and FRANI app took place simultaneously on each of the reference days, whereas the 24HR survey was undertaken the following day using the previous day as the reference period for food consumption. A team of 12 data collectors and 3 supervisors underwent 7 d of training using lecture, role-play, mock interview, and field practice methods. Trained enumerators visited participants early in the morning to hand the mobile phone to participants, and weigh and record food intake. On the following day, a different enumerator undertook the 24HR (7).

### FRANI mobile AI app

Standard model mobile phones were preloaded with a pre-configured FRANI AI app and provided to participants during a specified 7-d study period. Participants were trained and instructed to take pictures of the foods and beverages consumed at every meal, or instance of food consumption, using the FRANI mobile application. Users would take a picture of the meal they were about to consume, confirm the classification of food returned by FRANI, and input the amount of food actually consumed as the proportion of the total portion served. When FRANI image recognition was not accurate or the food item consumed was not part of the list of AI-recognized foods, users could record the particular food item consumed by selecting the appropriate item from a comprehensive list of foods consumed in Vietnam compiled from the Food Matters database (14). To facilitate the estimation of portion sizes, a “pop-socket” was used as a standardized visual prop when capturing images of food being consumed. A pop-socket is a small disc of standard size (1.56 inches in diameter) that the respondent placed next to the food when taking the picture of the food they were about to consume. The AI algorithm was designed to scale each pixel in the image using the pop-socket reference to estimate the 2-dimensional area covered by each food consumed and then estimate the weight in grams based on that area.

### Weighed records

Trained enumerators shadowed study participants on 3 nonconsecutive days from early morning until after the last evening meal, weighing and recording chronologically all foods and beverages that respondents ate at home, outside the home, or at school using digital scales (Tanita KD160, 2-kg capacity) accurate to 1 g. For each eating episode, the enumerator weighed each food or beverage before and after consumption. Enumerators recorded eating time, name of the food or recipe, eating place, weight of food, and leftover foods. If an item had residual waste (bones, skin, etc.), the enumerator recorded the weight of the waste so that this weight could be removed during data processing. Each day, enumerators asked participants to verify that no meals had been consumed before their arrival. If a meal had been eaten before the enumerator's arrival, the enumerator would record it using an alternative weighing method (by direct weight of the food if available, or by proxy weight with dry rice or water, or by using a photo book to estimate the quantity of food consumed). Before leaving in the evening, enumerators confirmed that participants had eaten their last meal of the day, checked that all FRANI records had been uploaded (i.e., each food had been input into FRANI), recorded having received the participant's confirmation, and departed.

### Multi-pass 24HR

Quantitative 24HRs were also conducted on 3 nonconsecutive days, on the day after the WR, by a different enumerator who had conducted the WR the previous day, using conventional paper-based data collection (7). During the first pass, the respondent was asked to list all of the foods and beverages including water that she had consumed the previous day from when she woke up in the morning until she went to bed. During the second pass, the respondent was asked to provide a detailed description of each food or beverage reported in the first pass. During the third pass, respondents were requested to provide an estimation of the quantity of each food or beverage consumed using portion-size estimation aids such as standard plates, bowls, cups, and other common household utensils. The 4 portion-size estimation methods included direct weight, proxy weight with dry rice, proxy weight with water, and photo book (100% and graduated images of food portions, expressed in grams). In the fourth pass, enumerators reviewed all of the foods and beverages with respondents to ensure that there were no omissions or intrusions of foods during the past 24 h.

### Data checking, cleaning, and processing

All FRANI pictures were checked every night by a study coordinator to make sure they had been uploaded correctly. Quality checks on paper forms for WR and 24HRs were conducted by field supervisors for completeness and correct food code correspondence to food descriptions. Data from the paper forms for the WR and 24HRs were entered into KoBoToolbox databases and checked for errors by double entry. Additional cleaning was conducted to check on food code, measurement method, and portion size. Food intakes for all 3 methods were converted to nutrient intakes using a 2007 Vietnamese food-composition table (15), including adjustment for nutrient retention of cooked foods (16, 17). The missing nutrient information for some foods was updated based on the food-composition database from the Vietnam General Nutrition Survey 2019–2020, Thai food-composition table (18), Asian food-composition table (19), and the USDA food data center (20).

For the adequacy of nutrient intake calculations, the usual intakes of micronutrients were estimated using the intra-person or intra-day variance method (21). Existing Stata syntax developed by the Women's Dietary Diversity Project (22) was adapted to calculate distributions of usual nutrient intakes and the probability of adequacy for 11 micronutrients (vitamin A, vitamin C, thiamin, riboflavin, niacin, vitamin B-6, vitamin B-12, folate, calcium, zinc, and iron). The Estimated Average Requirements (EARs) and SDs for age and sex were based on WHO/FAO recommendations (23), the International Zinc Nutrition Consultative Group recommendations for zinc (24), and Institute of Medicine recommendations for calcium (25), assuming low levels of bioavailability for iron (5%) and zinc (15%). The mean probability of adequacy (MPA) of micronutrient intake was calculated as the mean of the probability of adequacy for the 11 micronutrients.

### Statistical analysis

Descriptive analysis was conducted to report energy and nutrient intakes by person-day for each method. Nutrient intakes were tested for normality using Shapiro–Wilk tests. Because most distributions of nutrient intakes were skewed, we reported both mean (SD) and median (IQR) intakes. All nutrients were log-transformed for statistical testing.

Bland–Altman plots were used to depict the individual differences in intakes of energy and macro- and micronutrients by the 2 methods (WR-FRANI app or WR- 24HR) compared with the average intake by the 2 methods, respectively. Limits of agreement (LOA) were calculated as the mean difference ± 1.96 SDs, and interpreted as the range where 95% of differences were expected to occur (26).

Differences in log-transformed nutrient intake values between FRANI and WR, and between 24HR and WR methods, were calculated. The differences in log-transformed intakes are equivalent to the ratios of intake estimated by FRANI or 24HRs divided by the estimate WR. Mean differences by method were then estimated for each nutrient with regression models including random effects at the person level to account for repeated measures. The regressions provided the basis for equivalence testing (8, 27) using 10% (i.e., with 90% CI falling within a ratio of 0.9 to 1.1), 15% (i.e., with 90% CI falling within a ratio of 0.85 to 1.15), and 20% bounds (i.e., with 90% CI falling within a ratio of 0.8 to 1.2) based on validation studies in the literature (8, 27–29). The concordance correlation coefficient (CCC) estimated with adjustment for repeated measures was used to assess the extent of agreement between the 3 methods (30).

To identify sources of errors, we compared the proportion of adolescents who consumed each food group, the quantity consumed of each food group, and the percentage of energy intake from each food group by the 3 different methods. Food-group intake was categorized into 10 food groups as proposed by the Minimum Dietary Diversity for Women (MDD-W) guideline (31), including the following: *1*) grains, roots, and tubers; *2*) pulses; *3*) nuts and seeds; *4*) dairy products; *5*) meat, fish, and poultry; *6*) eggs; *7*) dark-green vegetables; *8*) vitamin A–rich fruits and vegetables; *9*) other vegetables; and *10*) other fruits. We also examined sources of errors by individual foods including *1*) the number of omissions (foods consumed but not reported) and intrusions (foods reported that were not consumed) for FRANI and 24HRs and *2*) portion estimation errors, which compared the mean of reported food amounts for the most commonly consumed foods (those with ≥10 consumption episodes) for FRANI and 24HRs with WR. Data were analyzed with STATA software version 16.0 (StataCorp) and R (R Core Team).

### Ethical approval

The study was approved by the Ethical Committee of Thai Nguyen National Hospital in Vietnam and the Institutional Review Board at the International Food Policy Research Institute, Washington, DC. Written informed assent and consent were obtained from all study participants and their caregivers.

## Results

On average, adolescent females were 14 y old (range: 12–18 y). All participants were attending middle or high school and ∼90% owned smartphones (**Table 1**). Participants lived in households of 4 people, on average, and more than 90% of households owned essential assets such as television, computer, refrigerator, air conditioners, washing machine, gas cooker, and motorbike. More than half of the adolescents’ parents had completed college or higher educational level and most of them (>80%) worked as white-collar workers or in service/sale areas.

**TABLE 1:** Characteristics of adolescents^1^

Characteristics	Values
Age, mean (SD), y	14.4 (1.9)
Having smart phone, %	88.9
Number of people in household, mean (SD)	4.3 (1.0)
Proportion of household owning assets, %	
Television	100.0
Computer	91.7
Refrigerator, freezer	100.0
Air conditioners	100.0
Washing machine	100.0
Gas cooker or stove	97.2
Water heater	75.0
Electric bicycle	50.0
Motorbike	94.4
Car	58.3
Proportion of participants with excellent school performance,^2^ %	
Math	24.0
Physics	12.5
Chemistry	27.8
Biology	28.0
Literature	8.3
History	33.3
Geography	20.8
Foreign language	15.4
Mother's level of education, %	
Less than high school	13.9
High school	33.3
College	33.3
Postgraduate (master's, PhD)	19.4
Father's level of education, %	
Less than high school	13.9
High school	30.6
College	36.1
Postgraduate (master's, PhD)	19.4
Mother's occupation, %	
Blue-collar worker	2.8
White-collar worker	38.9
Service, sale	41.7
Stay-at-home parent	2.8
Other	13.9
Father's occupation, %	
Blue-collar worker	8.3
White-collar worker	41.7
Service, sale	44.4
Stay-at-home parent	0.0
Other	5.6

1 *n* = 36.

2 School performance is subjectively assessed by midterm and final exams. Excellent school performance is defined as test score ≥9.

Overall daily intakes were low across all 3 methods (**Table 2**). Mean energy intakes were 1314, 1376, and 1344 kcal/d from WR, the FRANI app, and 24HRs, respectively. Mean and median intakes were lower than WHO-recommended nutrient intakes (23) for all nutrients except for vitamin C. The probability of adequate intake and MPA of nutrient intake were very low (<10% for all nutrients except for vitamin C at 16%; MPA ∼5–6%) for all 3 methods (**Table 3**).

**TABLE 2:** Nutrient intakes of adolescents by 3 d of observed weighed records, FRANI app, and 24-h recall^1^

	Weighed records (*n* = 108)^2^		FRANI app (*n* = 108)		24-h Recall (*n* = 108)	
	Mean (SD)	Median (IQR)	Mean (SD)	Median (IQR)	Mean (SD)	Median (IQR)
Energy, kcal	1314 (432)	1294 (592)	1376 (541)	1243 (810)	1344 (482)	1263 (680)
Protein, g	51.7 (20.7)	48.1 (26.1)	52.9 (22.7)	48.7 (26.8)	54.3 (23.5)	53.6 (30.7)
Fat, g	40.1 (19.6)	37.4 (26.9)	40.2 (23.2)	34.8 (25.9)	41.7 (21.6)	40.9 (31.3)
Carbohydrate, g	187.1 (69.4)	168.2 (87.5)	201.8 (87.9)	185.3 (115.3)	188.7 (74.8)	170.2 (97.3)
Fibre, g	4.6 (3.1)	4 (3.3)	4.8 (3.7)	4.1 (3.6)	4.4 (2.9)	4.2 (3.2)
Calcium, mg	373.5 (187.8)	325 (230.2)	356.9 (204.3)	288.5 (210.7)	365.0 (187.0)	315.2 (261.2)
Folate, μg	146.3 (97.5)	131.2 (122.2)	139.0 (98.5)	122.7 (108.3)	138.9 (97.3)	118.6 (115.7)
Iron, mg	9.9 (6.5)	8.8 (4.9)	9.9 (6.1)	8.5 (6)	9.6 (5.2)	8.7 (5.7)
Niacin, mg	8.2 (4.1)	7.8 (4.2)	8.6 (4.6)	8.2 (5)	9.0 (4.8)	7.8 (5.6)
Riboflavin, mg	0.6 (0.3)	0.6 (0.4)	0.6 (0.3)	0.6 (0.4)	0.6 (0.4)	0.6 (0.3)
Thiamin, mg	0.9 (0.4)	0.8 (0.5)	0.8 (0.5)	0.8 (0.7)	0.9 (0.5)	0.8 (0.6)
Vitamin A (RAE), μg	117.4 (130.3)	93.5 (126.3)	126.6 (160.7)	74.1 (142.6)	117.9 (131.1)	89.9 (134.5)
Vitamin B-6, mg	0.9 (0.4)	0 (0)	0.9 (0.4)	0 (0)	1.0 (0.8)	0 (0)
Vitamin B-12, μg	1.8 (2.0)	0.8 (0.4)	1.6 (1.5)	0.8 (0.4)	1.9 (2.1)	0.9 (0.6)
Vitamin C, mg	55.3 (43.0)	1.4 (1.3)	61.6 (50.6)	1.3 (1.4)	52.5 (35.7)	1.4 (1.4)
Zinc, mg	7.4 (3.2)	46.5 (50.2)	7.8 (3.7)	46.2 (64.2)	7.7 (3.6)	48.6 (48.1)

1 FRANI, Food Recognition Assistance and Nudging Insights; RAE, retinol activity equivalents.

2 Number of person-days = 108, equal to number of subjects (=36) multiplied by number of recalls (=3).

**TABLE 3:** Probability of adequate intake among adolescents by 3 d of weighed records, FRANI app, and 24-h recall^1^

	EAR^2^	Weighed records (*n* = 108),^3^ %	FRANI app (*n* = 108), %	24-h Recall (*n* = 108), %
Calcium, mg	1100	0.0	0.0	0.0
Iron, mg	20.5–28.4	3.8	3.5	1.9
Zinc, mg	7.0–8.8	8.0	9.0	8.9
Vitamin A (RAE), μg	365	0.6	0.1	0.4
Vitamin C, mg	33	15.0	16.6	16.5
Thiamin, mg	0.9	9.1	8.2	11.7
Riboflavin, mg	0.8	4.7	4.5	4.6
Niacin, mg	12	2.2	3.2	4.4
Vitamin B-6, mg	1.0	6.7	6.3	8.8
Folate, μg	330	0.5	0.8	0.7
Vitamin B-12, μg	2.0	6.3	4.4	6.8
Mean probability of adequacy of micronutrients, %		5.2	5.2	5.9

1 EAR, Estimated Average Requirement; FRANI, Food Recognition Assistance and Nudging Insights; IOM, Institute of Medicine; IZiNCG, International Zinc Nutrition Consultative Group; RAE, retinol activity equivalents.

2 The EAR and SDs for age and sex were based on WHO/FAO recommendations (23) for all nutrients, except for the IZiNCG recommendations for zinc (24), and IOM recommendations for calcium (25), assuming low levels of bioavailability for iron (5%) and zinc (15%).

3 Number of person-days = 108, equal to number of subjects (=36) multiplied by number of recalls (=3).

The distributions of energy and nutrients estimated by the FRANI app compared with WR are displayed in Bland–Altman plots (**Supplemental Figure 1**), with 95% of differences in intakes expressed as a ratio for log-transformed data. The LOA were narrow (<1) for energy and most nutrients except for vitamins A, B-12, and C and folate. The proportion falling outside the LOA was <10%.

**FIGURE 2:**
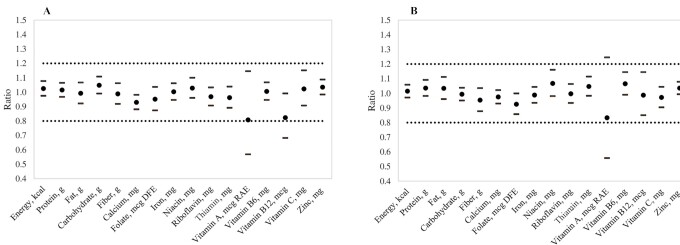
Equivalence testing of ratios of nutrient intake on 3 d by weighed records, FRANI app, and 24-h recall. (A) FRANI app/weighed records. (B) Twenty-four-hour recall/weighed records. Values are means and 95% CIs for test of equivalence (*n* = 36). Dotted lines showed 20% equivalence bounds. Note: (1 − ratio) × 100 is equal to the % error, and ratios between 0.9 and 1.1 are equivalent to a 10% bound around the mean % error. A 90% CI is used because 2 one-sided tests are performed (each with α of 0.05). The ratio is back-transformed from the difference in the log-FRANI nutrient minus the log-weighed record nutrient intake or log-24-h recalled nutrient minus the log-weighed record nutrient intake. Mean differences by method were estimated for each nutrient with regression models including random effects at the person-level to account for repeated measures. DFE, dietary folate equivalents; FRANI, Food Recognition Assistance and Nudging Insights; RAE, retinol activity equivalents.

**FIGURE 3:**
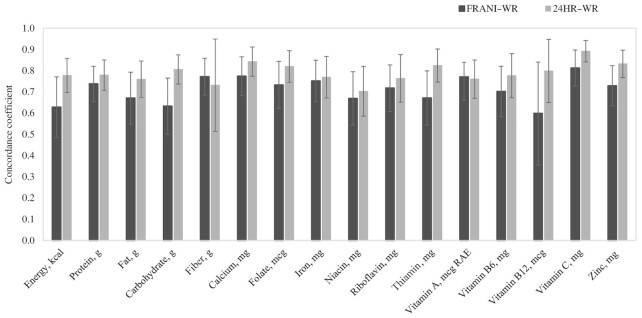
Concordance correlation coefficients of nutrient intakes on 3 d by WR, FRANI app, and 24HR (*n* = 36). The concordance correlation coefficient (CCC) was estimated with adjustment for repeated measures. FRANI, Food Recognition Assistance and Nudging Insights; RAE, retinol activity equivalents; WR, weighed records; 24HR, 24-h recall.

Relative differences between FRANI and WR were assessed by ratios of log-transformed intakes from FRANI to WR (**Figure 2**A and **Supplemental Table 1**). When comparing FRANI with WR, mean energy, protein, and fat intakes were equivalent at the 10% bound, with a mean ratio of 1.02 (90% CI: 0.98, 1.08) for energy, 1.02 (90% CI: 0.97, 1.07) for protein, and 0.99 (90% CI: 0.92, 1.07) for fat. FRANI/WR ratios for all micronutrients were within a 20% bound, except for vitamins A and B-12; equivalence ratios were within a 15% bound for 3 nutrients (calcium, niacin, and thiamin) and within a 10% bound for 4 nutrients (iron, riboflavin, vitamin B-6, and zinc). Similar results were observed for differences between 24HRs and WR, with estimates falling within 20% equivalence bounds for all nutrients except for vitamin A (Figure 2B and Supplemental Table 1). The CCCs by nutrient between FRANI and WR ranged between 0.60 and 0.81, with slightly higher CCCs found for 24HRs and WR (ranging between 0.70 and 0.89). Differences, however, were not statistically significant, as shown by the overlap of 95% CIs (**Figure 3** and **Supplemental Table 2**).

**FIGURE 4:**
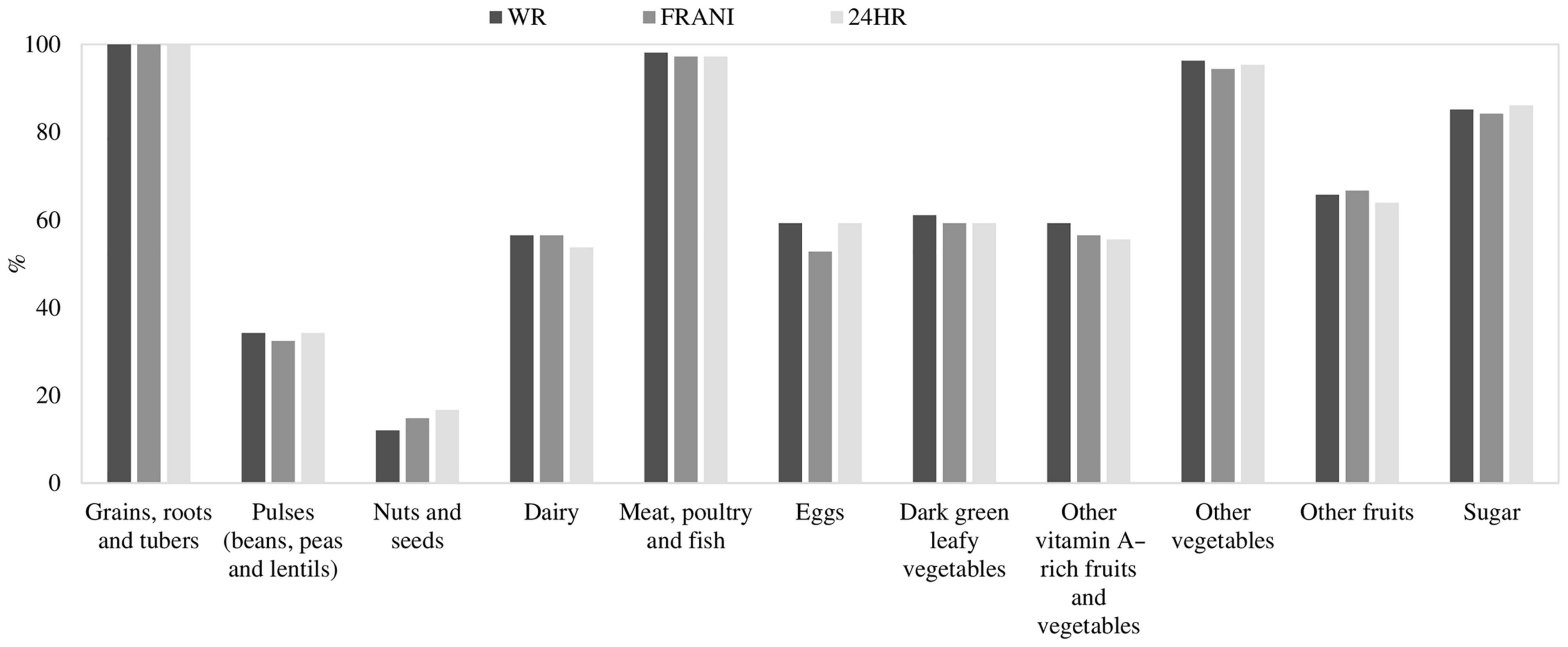
Food groups consumed by WR, FRANI app, and 24HR (*n* = 36). The mean number of food groups consumed was 6.42 ± 1.52 for WR, 6.31 ± 1.58 for FRANI, and 6.35 ± 1.52 for 24HRs. The proportion of adolescents who consumed at least 5 food groups was 89.8% for WR, 85.2% for FRANI, and 88% for 24HRs. FRANI, Food Recognition Assistance and Nudging Insights; WR, weighed records; 24HR, 24-h recall.

The proportion of days when adolescents consumed different food groups was similar among the 3 methods (**Figure 4**). While almost all adolescents (94–100%) consumed grains, meat, and other vegetables daily, only one-third consumed pulses and 12–17% consumed nuts and seeds. Approximately half of them consumed dairy, egg, fruit, and vegetables. On average, adolescents consumed ∼6 food groups each day and more than 80% consumed at least 5 food groups per day.

When examining the share of estimated energy intakes consumed by major food groups (**Table 4**), there was some variation in mean and median quantities consumed for both FRANI and 24HR methods when compared with WR. FRANI tended to overestimate consumption from grains, meat, other vegetables, and fruits, but underestimate consumption from other food groups. 24HRs overestimated consumption of pulses, nuts and seeds, meat, and vegetables and underestimated consumption of eggs, dark-green leafy vegetables, and other fruits. When comparing FRANI with WR, the levels of omission and intrusion errors were both found to be at 21%. Omission and intrusion errors were slightly lower when comparing 24HRs and WR (16% and 19%, respectively). Errors in portion estimation for the most commonly consumed foods by different methods are presented in **Table 5**, showing evidence of no clear bias direction in portion-size estimation by specific food.

**TABLE 4:** Quantity of food group consumed and % of energy intake from major food groups on 3 d by weighed records, FRANI app, and 24-h recall^1^

	Weighed records (*n* = 108)^2^		FRANI app (*n* = 108)		24-h Recall (*n* = 108)	
	Mean (SD)	Median	Mean (SD)	Median	Mean (SD)	Median
Quantity consumed, g						
Grains, roots, and tubers	209.2 (96.4)	185.7	230.6 (125.2)	203.2	209.9 (108.6)	186.1
Pulses (beans, peas, and lentils)	37.2 (87.7)	0.0	36.0 (96.8)	0.0	41.6 (97.5)	0.0
Nuts and seeds	1.1 (4.2)	0.0	1.1 (3.1)	0.0	4.9 (26.0)	0.0
Dairy	97.1 (112.2)	62.1	77.8 (92.2)	36.0	89.6 (110.6)	23.7
Meat, poultry, and fish	121.5 (83.2)	107.7	133.7 (110.0)	104.6	153.3 (194.3)	118.4
Eggs	30.4 (41.7)	13.1	22.1 (29.0)	7.2	26.7 (38.2)	14.6
Dark-green leafy vegetables	31.9 (45.6)	10.8	24.5 (32.3)	8.5	30.3 (42.4)	7.7
Other vitamin A–rich fruits and vegetables	26.0 (49.1)	2.2	22.2 (39.7)	1.4	28.2 (55.3)	1.6
Other vegetables	86.8 (90.6)	65.9	98.8 (109.7)	60.0	87.7 (89.4)	65.1
Other fruits	58.3 (83.7)	23.4	80.0 (115.5)	32.6	53.3 (97.0)	12.4
Sugar	134.6 (128.1)	124.5	112.2 (113.1)	105.7	130.7 (134.0)	94.1
Percentage of energy intake, %						
Grains, roots, and tubers	54.1 (15.4)	54.8	55.9(15.5)	57.7	52.0 (14.9)	52.3
Pulses (beans, peas, and lentils)	3.1 (6.2)	0.0	2.6 (5.4)	0.0	3.0 (5.8)	0.0
Nuts and seeds	0.4 (1.5)	0.0	0.4 (1.2)	0.0	0.9 (3.2)	0.0
Dairy	5.8 (7.6)	2.7	4.4 (6.1)	1.8	5.2 (7.4)	0.4
Meat, poultry, and fish	16.0 (11.4)	13.4	17.4 (12.1)	15.4	17.8 (11.7)	15.9
Eggs	3.9 (5.9)	1.8	3.0 (4.4)	0.8	4.0 (6.7)	2.0
Dark-green leafy vegetables	0.8 (1.2)	0.3	0.6 (0.9)	0.3	0.7 (1.2)	0.2
Other vitamin A–rich fruits and vegetables	0.9 (2.2)	0.1	0.6 (1.4)	0.0	0.8 (1.9)	0.0
Other vegetables	2.9 (4.0)	1.9	2.8 (3.3)	1.8	2.3 (2.1)	1.9
Other fruits	2.6 (3.1)	1.5	3.4 (4.5)	2.0	2.5 (3.7)	0.8
Sugar	11.6 (10.8)	9.3	8.7(8.3)	6.5	11.4 (10.8)	9.0

1 FRANI, Food Recognition Assistance and Nudging Insights.

2 Number of person-days = 108, equal to number of subjects (=36) multiplied by number of recalls (=3).

**TABLE 5:** Sources of errors by 3 d of weighed records, FRANI app, and 24-h recall^1^

	Number of consumption episodes	Quantity, mean (SD), g			Ratio	
		Weighed records	FRANI app	24-h Recall	FRANI app/weighed records	24-h Recall/weighed records
Rice regular white, boiled	167	139.9 (56.0)	167.9 (81.6)	158.6 (69.9)	1.20	1.13
Broth of vegetable soup without oil	36	116.4 (54.3)	191.6 (79.0)	152.2 (67.9)	1.65	1.31
Chocolate drink packaged	24	187.1 (46.7)	143.9 (35.4)	187.8 (46.8)	0.77	1.00
Chicken meat, local, average, boiled	23	60.5 (38.9)	62.8 (29.5)	39.8 (30.6)	1.04	0.66
Sauce of mixed roll by Vietnamese rice paper	17	38.9 (66.6)	51.7 (24.5)	25.9 (27.4)	1.33	0.67
Red pepper sauce concentrate	16	11.3 (9.9)	41.6 (24.2)	17.0 (16.1)	3.70	1.51
Spring roll, pork, fried	16	92.7 (48.7)	113.8 (74.5)	97.1 (74.2)	1.23	1.05
Water spinach, boiled	16	84.6 (65.1)	65.2 (27.8)	74.6 (30.3)	0.77	0.88
Fish sauce, ready-to-serve	15	4.9 (2.6)	20.8 (14.4)	9.5 (3.4)	4.24	1.95
Longan	15	80.5 (48.3)	256.5 (89.9)	67.8 (39.7)	3.19	0.84
Hen egg, fried	14	50.9 (27.5)	52.4 (16.2)	49.9 (39.2)	1.03	0.98
Minced meat, pork, cooked	13	51.5 (19.9)	33.8 (18.8)	56.8 (31.3)	0.66	1.10
Cabbage, white/green, boiled	12	117.2 (67.8)	122.1 (57.5)	85.3 (37.9)	1.04	0.73
Yogurt, thick, with sugar, whole, white	12	94.8 (7.4)	90.9 (20.2)	100.7 (13.2)	0.96	1.06
Wax gourd, ash gourd, cooked, soup	11	163.5 (65.8)	116.9 (27.0)	98.6 (50.5)	0.71	0.60
Chicken, local, dry-stirred	11	45.8 (29.8)	67.7 (29.5)	62.1 (64.5)	1.48	1.36
Sugar apple	11	115.9 (19.8)	124.6 (22.6)	95.3 (17.9)	1.07	0.82
Whole hen egg, fried	11	56.5 (22.7)	51.0 (6.7)	32.5 (5.1)	0.90	0.58

l FRANI, Food Recognition Assistance and Nudging Insights.

## Discussion

To our knowledge, this study is one of very few to rigorously address an important evidence gap on the relative validity of using innovative AI-based mobile technology to assess the diets of adolescent females in LMICs. By comparing the FRANI app against the gold standard of WR and a traditional 24HR, our study showed that both the AI-assisted dietary assessment and the 24HRs accurately estimate energy and protein intake in adolescent females. For both FRANI and 24HR methods, equivalence was also determined for most nutrients at 15% bounds. Sources of errors mainly involved the limited recipes available for selection in FRANI, adolescents’ ability to accurately recall consumed foods, and portion-size estimation. Although adequacy of micronutrient intake was very low, no differences were found in the estimates across the 3 methods.

Dietary assessment methods that use mobile technology have been increasingly applied in nutritional studies in an effort to improve the availability and quality of dietary data. A current systematic review and meta-analysis (32) of validation studies examining mobile phone–based dietary assessment apps reported 14 studies, all of which were conducted in high-income countries and only 2 validated in adolescents [in Korea (33) and Sweden (34)]. Findings from this meta-analysis revealed that apps involving dietary records slightly underestimated food consumption compared with traditional dietary assessment methods (–85 kcal/d for energy; −19 g/d, −13 g/d, and −12 g/d for carbohydrate, fat, and protein intake, respectively) (32). Most of these studies, however, used 24HRs as the only reference method and only 2 used WR (35, 36). Our study used both WR and 24HRs as standard and reference methods, respectively, and did not find differences in energy and macronutrient intakes across 3 methods, but found similar patterns of underestimation for micronutrients (calcium, folate, vitamin A, and vitamin B-12) as reported in other adolescent studies (8, 33). One possible explanation for similar energy and macronutrient intake levels across the methods used could be due to the controlled study environment, where the enumerators who conducted the WR also reminded adolescents to take pictures before eating and to upload them in a timely manner. In addition, the WR and FRANI pictures could increase the salience of the consumption episodes the previous day and thus facilitate the 24HR process and accuracy.

The wide bounds observed for vitamins A and B-12 were likely due to a combination of estimation errors and large variance in the actual intake for these nutrients (partly due to the small sample size). For both nutrients, there was a low frequency of consumption of foods with extremely high nutrient content, leading to extreme values skewing the relevant nutrient intake distribution, including embryo duck eggs, pig liver, egg yolk, chicken giblets, cheese, paste for vitamin A and salmon, pig liver, chicken giblets, and egg yolk for vitamin B-12. With regard to estimation errors for vitamin A, this could involve *1*) lower portion-size estimation of eggs and vitamin A–rich fruit such as ripe mango, cantaloupe, and jackfruit or *2*) limitations of FRANI in capturing vitamin A–rich fruit and vegetables. In fact, there is some evidence in the literature suggesting that the reliability of fruit and vegetable intakes in validation studies tends to be low (35). For vitamin B-12, estimation errors could involve the underestimation of eggs and dairy by FRANI (although median meat intakes were similar) or its inability to pick up vitamin B-12–rich foods in a mixed dish. Further improvements in FRANI's food recognition and portion-size estimation, including foods rich in vitamins A and B-12 are currently underway, involving expanded image libraries to improve food recognition and the development of more sophisticated portion-estimation models, including use of depth information to estimate food volumes.

Although the potential for FRANI to accurately estimate food and nutrient intake and provide a basis for high-frequency, real-time dietary assessment is clear, some practical, usability constraints were also apparent. Users needed to be trained on how to use FRANI and take pictures appropriately, then selecting foods, adjusting portion sizes, and confirming the portions of the food they eat. In addition, users need to remember and actually take time to interact with FRANI during their mealtimes, which may inconvenience others who eat at the same table and, as in the case of Vietnam, from common food containers. Last, as the current AI component of FRANI is only able to recognize 255 highly popular foods, users need to manually select other foods names from a drop-down list of Vietnamese foods, a task that is prone to introduce errors, including omitting the food or choosing the wrong food names. Detailed analysis of the usability and acceptability of a FRANI pilot is also currently underway (37).

The strengths of this study include the rigorous methods involved in the validation exercise. Both WR and 24HRs were used as methods for comparison with FRANI, and data for these methods were collected by different enumerators to avoid any bias during data collection. The dietary data were also collected on 3 nonconsecutive days for each participant, including both weekdays and weekends, and were thus representative for usual intake based on different days of the week. We also acknowledge some important limitations of this analysis. First, the use of FRANI to record food intake was conducted in a relatively controlled environment, where enumerators were able to verify that FRANI was being used correctly, leading to increased precision in the FRANI estimation and results that may be more promising than in real-world situations. However, the accuracy of the 24HR is likely to be biased towards higher precision, as respondents were primed by the visual records in FRANI they had recorded the previous day. Hence, the relative comparisons suggesting equivalence between FRANI and 24HRs may still hold in a real-world setting, although this will be an important area of future research. Second, the sample size for this study was small but in line with expectations for a pilot (38), and participants were recruited using a convenience sampling method, which may limit generalizability, particularly with regard to the high literacy levels of the study participants. The ongoing parallel study in Ghana, involving a random population-based sample of adolescent females, will provide more insights on this particular point. Last, because WR enumerators could not shadow participants for the full 24-h recall period, there were some instances when participants consumed food when the enumerators were not present. To overcome this challenge, enumerators strived to reach the adolescents’ home as early as possible in the morning and to leave the home as late as possible in the evening. In the analysis, we also matched the times of observation to the recalls and excluded food items reported on the recall that were consumed before or after the WR.

In conclusion, both FRANI AI-assisted dietary assessment and 24HRs accurately estimate nutrient intake in adolescent females in Vietnam when compared with WR, the gold standard for dietary assessment (39). Errors could be reduced with improvements in AI-assisted food-recognition and portion estimation. Further research is underway, including a feasibility assessment of using FRANI to nudge adolescent females towards healthy food choices. Although the potential for impact of FRANI at scale is clear, real-world validation and feasibility assessments, as well as effectiveness studies, will be required to ensure that the technology development results in an intervention that is appropriate, valid, and effective. If successfully evaluated in a real-world setting, FRANI may provide important advances in real-time dietary assessment methods and an invaluable resource of high-frequency dietary data to improve diets and nutrition of adolescent females in LMICs.

## Supplementary Material

nqac216_Supplemental_File

## Data Availability

Data described in the manuscript, code book, and analytic code will be made available upon request.

## Notes

Author disclosures: The authors report no conflicts of interest.

Funding for this research was provided by Foundation Botnar. The funders had no role in study design, data collection and analysis, data interpretation, decision to publish, or preparation of the manuscript.

Supplemental Figure 1 and Supplemental Tables 1 and 2 are available from the “Supplementary data” link in the online posting of the article and from the same link in the online table of contents at https://academic.oup.com/ajcn/.

Abbreviations used: AI, artificial intelligence; CCC, concordance correlation coefficient; FRANI, Food Recognition Assistance and Nudging Insights; LMIC, low-and middle-income country; LOA, limits of agreement; MPA, mean probability of adequacy; WR, weighed records; 24HR, 24-h recall.
