# Supplementary material for: Relative validity of a mobile AI-technology–assisted dietary assessment in adolescent females in Vietnam
Source: Am J Clin Nutr. 2022 Aug 9;116(4):992–1001. doi: 10.1093/ajcn/nqac216 (PMC9535545; doi:10.1093/ajcn/nqac216)
Supplement: nqac216_Supplemental_File [file nqac216_supplemental_file.docx]

**ONLINE SUPPORTING MATERIALS**

**Supplemental Table 1: Equivalence testing of ratios of nutrient intakes on 3 days by weighed records, FRANI app and 24-h recall**

**Supplemental Table 2: Concordance coefficients of nutrient intakes on 3 days by weighed records, FRANI app and 24-h recall**

**Supplemental Figure 1: Bland-Altman plots depicting difference between estimate between weighed records and FRANI app methods**

**Supplemental Table 1: Equivalence testing of ratios of nutrient intakes on 3 days by weighed records, FRANI app and 24-h recall**

|  | **FRANI/ WR**  **(n=108)^1^** | | | | | | **24HR/ WR**  **(n=108)** | | | | | |
| --- | --- | --- | --- | --- | --- | --- | --- | --- | --- | --- | --- | --- |
|  | **Ratio** | **90% CI for test of equivalence of ratio** | | **Equivalence** | | | **Ratio** | **90% CI for test of equivalence of ratio** | | **Equivalence** | | |
|  | **Mean** | **Lower** | **Upper** | **10% bound** | **15% bound** | **20% bound** | **Mean** | **Lower** | **Upper** | **10% bound** | **15% bound** | **20% bound** |
| Energy, kcal | 1.02 | 0.98 | 1.08 | Yes | Yes | Yes | 1.01 | 0.97 | 1.06 | Yes | Yes | Yes |
| Protein, g | 1.02 | 0.97 | 1.07 | Yes | Yes | Yes | 1.04 | 0.98 | 1.09 | Yes | Yes | Yes |
| Fat, g | 0.99 | 0.92 | 1.07 | Yes | Yes | Yes | 1.03 | 0.96 | 1.11 | No | Yes | Yes |
| Carbohydrate, g | 1.05 | 0.99 | 1.11 | No | Yes | Yes | 0.99 | 0.95 | 1.04 | Yes | Yes | Yes |
| Fibre, g | 0.99 | 0.92 | 1.06 | Yes | Yes | Yes | 0.95 | 0.88 | 1.04 | No | Yes | Yes |
| Calcium, mg | 0.93 | 0.88 | 0.98 | No | Yes | Yes | 0.97 | 0.93 | 1.02 | Yes | Yes | Yes |
| Folate, mcg | 0.95 | 0.87 | 1.04 | No | Yes | Yes | 0.93 | 0.86 | 1.00 | No | Yes | Yes |
| Iron, mg | 1.00 | 0.95 | 1.06 | Yes | Yes | Yes | 0.99 | 0.94 | 1.04 | Yes | Yes | Yes |
| Niacin, mg | 1.03 | 0.96 | 1.10 | No | Yes | Yes | 1.07 | 0.98 | 1.16 | No | No | Yes |
| Riboflavin, mg | 0.97 | 0.91 | 1.03 | Yes | Yes | Yes | 1.00 | 0.93 | 1.06 | Yes | Yes | Yes |
| Thiamine, mg | 0.96 | 0.89 | 1.04 | No | Yes | Yes | 1.05 | 0.98 | 1.11 | No | Yes | Yes |
| Vitamin A (RAE), mcg | 0.81 | 0.57 | 1.15 | No | No | No | 0.83 | 0.56 | 1.25 | No | No | No |
| Vitamin B6, mg | 1.01 | 0.95 | 1.07 | Yes | Yes | Yes | 1.07 | 0.99 | 1.15 | No | Yes | Yes |
| Vitamin B12, mcg | 0.82 | 0.68 | 0.99 | No | No | No | 0.99 | 0.85 | 1.15 | No | Yes | Yes |
| Vitamin C, mg | 1.02 | 0.91 | 1.15 | No | No | Yes | 0.97 | 0.90 | 1.04 | Yes | Yes | Yes |
| Zinc, mg | 1.03 | 0.98 | 1.09 | Yes | Yes | Yes | 1.04 | 0.99 | 1.08 | Yes | Yes | Yes |

^1^Number of person-day. 24HR: 24-hour recall. CI: Confident interval, FRANI: Food Recognition Assistance and Nudging Insights, WR: Weighed records

Note: (1 − ratio) * 100 is equal to the percent error, and ratios between 0.9 and 1.1 are equivalent to a 10% bound around the mean percent error. A 90% confidence interval is used because two one-sided tests are performed (each with α of 0.05). ^1^The ratio is back-transformed from the difference in the log-FRANI nutrient minus the log-weighed record nutrient intake or log-24h recalled nutrient minus the log-weighed record nutrient intake.

**Supplemental Table 2: Concordance coefficients of nutrient intakes on 3 days by weighed records, FRANI app and 24-h recall**

|  | **FRANI and WR concordance**  **(n=108)^1^** | | | **24HR and WR concordance**  **(n=108)** | | |
| --- | --- | --- | --- | --- | --- | --- |
|  | **CCC** | **95% CI** | | **CCC** | **95% CI** | |
|  | **Mean** | **Lower** | **Upper** | **Mean** | **Lower** | **Upper** |
| Energy, kcal | 0.63 | 0.48 | 0.77 | 0.78 | 0.70 | 0.86 |
| Protein, g | 0.74 | 0.65 | 0.82 | 0.78 | 0.71 | 0.85 |
| Fat, g | 0.67 | 0.55 | 0.79 | 0.76 | 0.67 | 0.85 |
| Carbohydrate, g | 0.63 | 0.50 | 0.76 | 0.81 | 0.74 | 0.87 |
| Fibre, g | 0.77 | 0.68 | 0.86 | 0.73 | 0.51 | 0.95 |
| Calcium, mg | 0.77 | 0.68 | 0.87 | 0.84 | 0.77 | 0.91 |
| Folate, mcg | 0.73 | 0.62 | 0.84 | 0.82 | 0.75 | 0.89 |
| Iron, mg | 0.75 | 0.65 | 0.85 | 0.77 | 0.67 | 0.87 |
| Niacin, mg | 0.67 | 0.54 | 0.79 | 0.70 | 0.59 | 0.82 |
| Riboflavin, mg | 0.72 | 0.61 | 0.83 | 0.76 | 0.65 | 0.88 |
| Thiamine, mg | 0.67 | 0.54 | 0.80 | 0.82 | 0.75 | 0.90 |
| Vitamin A, mcg RAE | 0.77 | 0.66 | 0.84 | 0.76 | 0.65 | 0.85 |
| Vitamin B6, mg | 0.70 | 0.58 | 0.82 | 0.78 | 0.67 | 0.88 |
| Vitamin B12, mcg | 0.60 | 0.36 | 0.84 | 0.80 | 0.65 | 0.95 |
| Vitamin C, mg | 0.81 | 0.73 | 0.90 | 0.89 | 0.84 | 0.94 |
| Zinc, mg | 0.73 | 0.63 | 0.82 | 0.83 | 0.77 | 0.90 |

^1^Number of person-day. 24HR: 24-hour recall. CCC: Concordance correlation coefficient, CI: Confident interval, FRANI: Food Recognition Assistance and Nudging Insights, WR: Weighed records

**Supplemental Figure 1: Bland-Altman plots depicting difference between estimate between weighed records and FRANI app methods**

| **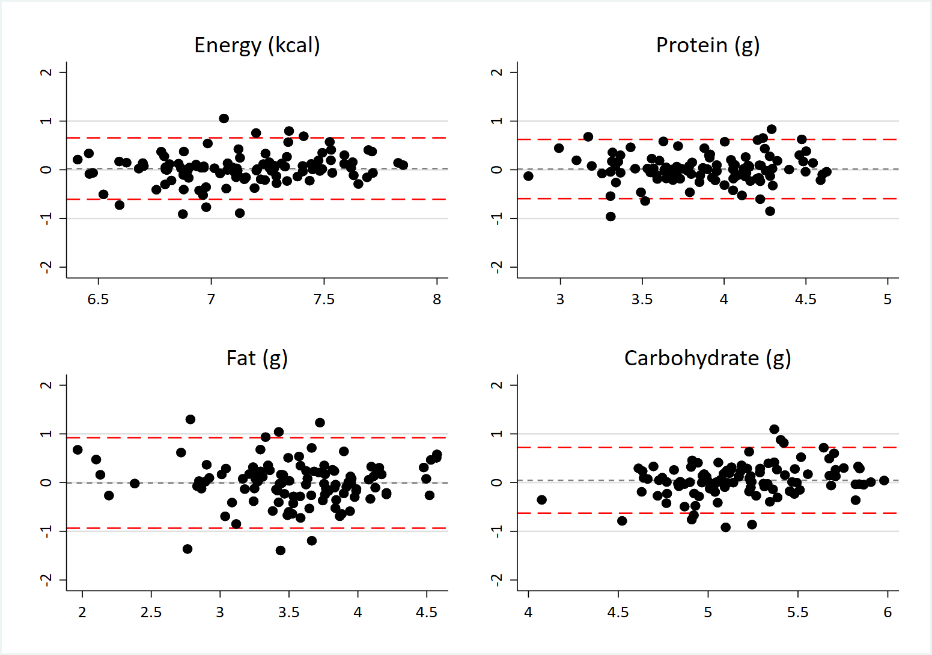** | **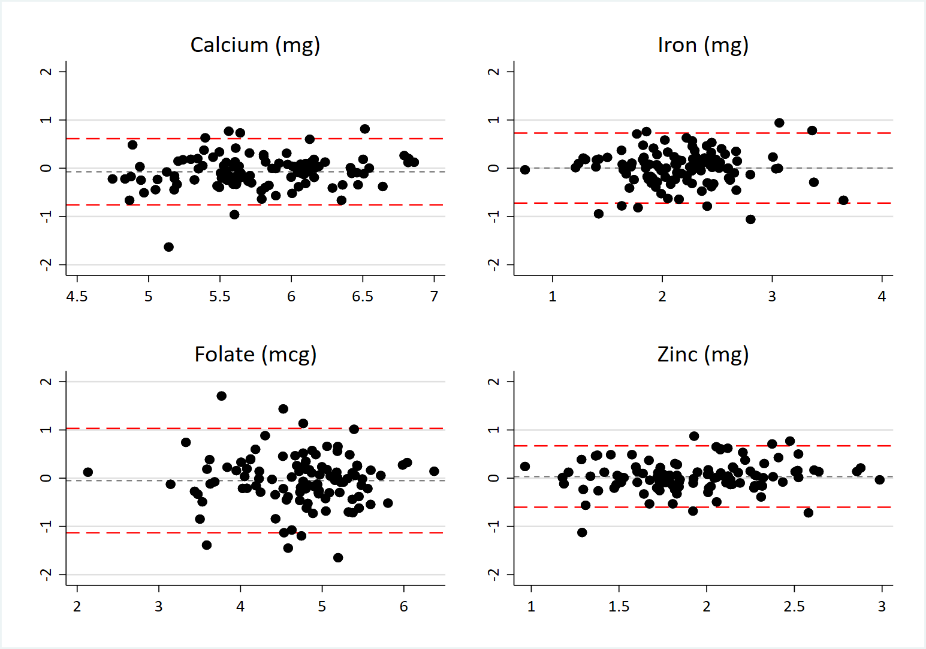** |
| --- | --- |
| **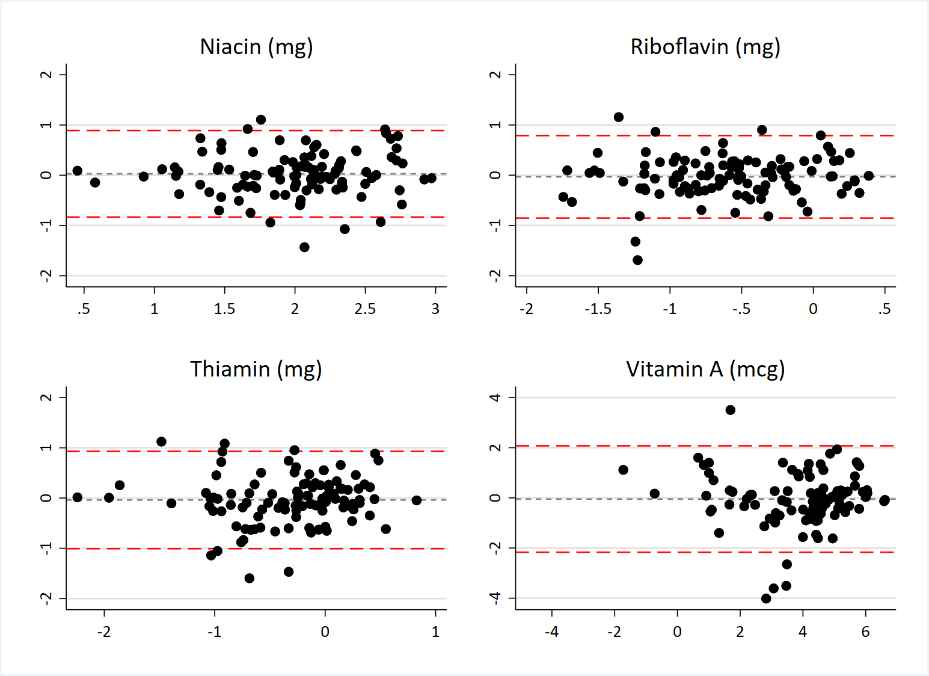** | **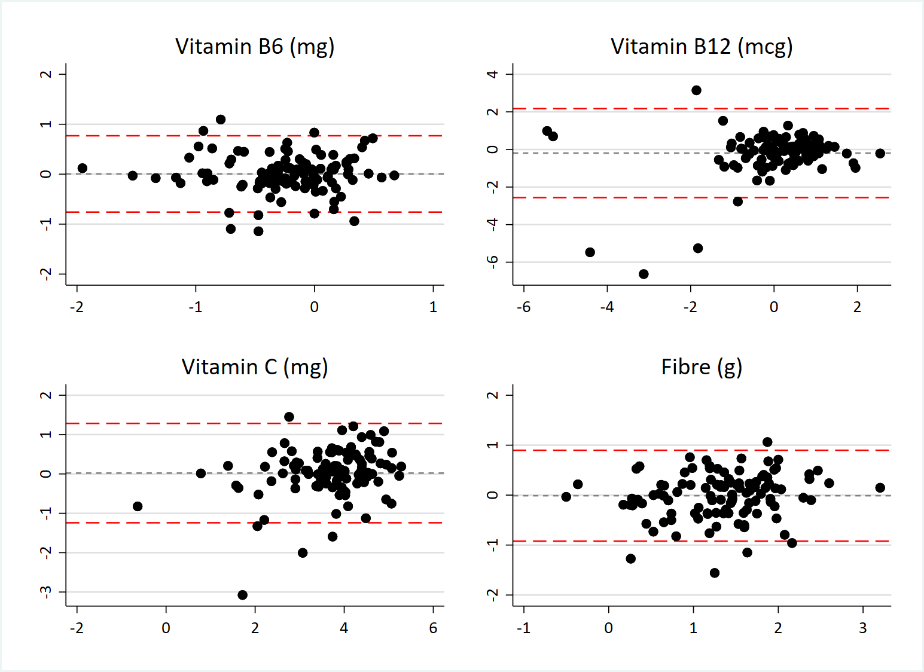** |

FRANI: Food Recognition Assistance and Nudging Insights. The grey dotted horizontal line is at the mean difference in nutrient intake calculated as FRANI minus Weighed record nutrients, expressed as a ratio for log-transformed data. The red dashed lines are the upper and lower lines of agreement (mean +/- 1.96 SD) containing 95% of values.
